# Soil temperature and respiration dataset from Mt. Namsan: Long-term projection and its implications under SSP climate scenarios

**DOI:** 10.1016/j.dib.2025.111397

**Published:** 2025-02-18

**Authors:** Jae-Ho Lee, Young-Ju Yu, Yong-Su Kwon, Jae-Seok Lee

**Affiliations:** a Ecological Information Team, National Institute of Ecology, Seocheon, 33657, Republic of Korea; b Climate Change Response Team, Korea Agriculture Technology Promotion Agency, Iksan 54667, Republic of Korea; c Department of Biological Sciences, Konkuk University, Seoul, 05029, Republic of Korea

**Keywords:** Forest carbon fluxes, Climate change modeling, Soil carbon dynamics, Regression-based predictions, SSP climate scenarios

## Abstract

This study presents a comprehensive dataset of soil temperature and respiration measurements from Mt. Namsan, South Korea, collected between 2009 and 2010, with estimated values for 2011 to 2020 and projections for 2021 to 2100 under Shared Socioeconomic Pathways (SSP) climate scenarios ranging from SSP1-2.6 to SSP5-8.5. Soil respiration (mg CO₂ m⁻² h⁻¹) and temperature (°C) were measured using automated sensors and the closed chamber method. Regression models were developed to estimate soil temperature for 2011–2020 and predict future trends based on SSP scenarios. The results demonstrate significant increases in soil respiration rates under SSP5-8.5, highlighting the temperature sensitivity of soil carbon fluxes. This dataset provides valuable insights for ecological modeling, long-term carbon budget analysis, and climate change adaptation studies in temperate forest ecosystems.

Specifications Table

**Table:** 

Subject	Ecology, Climate Science
Specific subject area	Soil respiration, climate scenarios, carbon cycling
Type of data	Tables, Figure
Data collection	Data were collected using sensors (Testo-925 temperature sensor and Vaisala GMT-222/343 CO₂ sensor) at a depth of 5 cm. Monthly averages of soil temperature (°C) and soil respiration (mg CO₂ m⁻² h⁻¹) were recorded between 2009 and 2010 using automated systems. CO₂ concentration was measured using the closed chamber method. Future climate scenario data (SSP1-2.6, SSP2-4.5, SSP3-7.0, SSP5-8.5) were sourced from the Korea Meteorological Administration (http://www.climate.go.kr/), and air temperature data were used to predict soil temperature and respiration through regression models.
Data source location	Latitude: 37°32′53.5″ N, Longitude: 126°59′40.1″ E, Mt. Namsan, South Korea
Data accessibility	Repository name: ecobank Data identification number: http://doi.or.kr/10.22756/ETC.20250000000916 Direct URL to data: http://doi.or.kr/10.22756/ETC.20250000000916 Instructions for accessing these data: To access the dataset, users should follow the direct URL provided above. No special permissions are needed for download.
Related research article	Not applicable

## 1 Value of the Data

• **Comprehensive dataset**:
  This dataset offers a unique combination of measured, estimated, and projected soil temperature and respiration data from Mt. Namsan, South Korea. It spans over a century (2009–2100) and is based on Shared Socioeconomic Pathways (SSP) climate scenarios ranging from SSP1-2.6 to SSP5-8.5. This extended temporal coverage makes it invaluable for understanding long-term trends in soil carbon fluxes.
• **Applicability to ecological modeling**:
  The data provide critical inputs for validating and improving ecological models, particularly those related to carbon cycling and temperature-driven processes in temperate forests. Researchers can leverage this dataset to simulate future soil respiration dynamics under different climate change scenarios.
• **Broad applications**:
  This dataset is particularly suited for:
  - Large-scale spatial assessments of soil carbon dynamics.
  - Climate impact modeling to evaluate the effects of varying emissions pathways.
  - Identification of drivers of soil respiration, enabling the analysis of key factors influencing carbon fluxes.
• **Scientific significance**:
  The dataset bridges critical knowledge gaps by offering robust measurements alongside future projections. This integration of empirical and modeled data enhances our ability to predict the response of temperate forest ecosystems to global warming.
• **Public accessibility**:
  The dataset is openly available, enabling reuse across disciplines such as climate science, ecology, and environmental management. It serves as a reproducible resource for conducting comparative studies and refining climate adaptation strategies.

## 2 Background

Soil respiration is a fundamental component of the global carbon cycle and represents one of the largest pathways for CO₂ exchange between the soil and the atmosphere. It is primarily influenced by environmental factors such as soil temperature, moisture, and vegetation type, with soil temperature being a critical determinant of respiration rates across various ecosystems [1,2]. Understanding how soil respiration responds to temperature changes is essential for predicting carbon fluxes and modeling the impacts of global climate change on forest ecosystems [[3], [4]].

Despite considerable research, significant knowledge gaps remain in understanding the long-term dynamics of soil respiration under diverse climate scenarios. Many studies have focused on short-term measurements or specific regions, which limits their applicability to larger spatial and temporal scales. Furthermore, few datasets integrate measured, estimated, and projected values, which are critical for examining soil respiration dynamics over time [5]. The lack of such comprehensive datasets has hindered the development of robust models to predict future carbon fluxes and evaluate the effects of climate change.

To address these gaps, this study compiles a long-term dataset of soil temperature and respiration from the *Quercus mongolica* forest at Mt. Namsan, South Korea. The dataset includes measured data from 2009 to 2010, estimated data for 2011 to 2020 using regression models, and projected data for 2021 to 2100 under the Shared Socioeconomic Pathway (SSP) climate scenarios ranging from SSP1-2.6 to SSP5-8.5. This comprehensive approach integrates empirical observations with future climate predictions, providing a valuable resource for analyzing soil carbon dynamics.

The primary aim of this study is to investigate the relationship between soil temperature and respiration across multiple timescales and under varying emission pathways. Specifically, we seek to address the following research questions:

1) How does soil respiration respond to temperature variations under different SSP scenarios?
2) Does the sensitivity of soil respiration differ significantly between low-emission (SSP1-2.6) and high-emission (SSP5-8.5) pathways?

We hypothesize that soil respiration rates will exhibit a temperature-dependent exponential increase, with the greatest sensitivity observed under high-emission scenarios such as SSP5-8.5. By combining measurements, estimates, and projections, this study offers new insights into the long-term impacts of climate change on temperate forest ecosystems and supports the development of effective adaptation strategies. The findings contribute to closing critical knowledge gaps in carbon cycle modeling and ecological responses to climate change [6,7].

## 3 Data Description

The dataset provides monthly average soil temperature and respiration data categorized into three distinct periods: Measured Data (2009–2010), Estimated Data (2011–2020), and Projected Data (2021–2100). Each category is described below:

1. **Measured Data (2009-2010)**
  This category contains direct measurements of soil temperature (°C) and respiration rates (mg CO₂ m⁻² h⁻¹) recorded at a depth of 5 cm in the Quercus mongolica forest at Mt. Namsan, South Korea. Measurements were obtained using the Testo-925 temperature sensor and Vaisala GMT-222/343 CO₂ sensor with the closed chamber method. Monthly averages were calculated from raw data collected continuously between January 2009 and December 2010. These data serve as the empirical foundation for subsequent regression models and future projections.
2. **Estimated Data (2011-2020)**
  Since direct measurements were unavailable for this period, soil temperature was estimated using a linear regression model developed from the measured data (2009–2010) and air temperature data from the Korea Meteorological Administration (KMA). The regression equation relates air temperature to soil temperature at the study site, ensuring reliable estimations. Soil respiration data for this period were not estimated due to insufficient observational data for validation. This estimated dataset bridges the gap between measured and projected data, enabling a more continuous temporal analysis.
3. **Projected Data (2021-2100)**
  This category includes soil temperature and respiration projections under Shared Socioeconomic Pathway (SSP) scenarios: SSP1-2.6, SSP2-4.5, SSP3-7.0, and SSP5-8.5. Projections were generated using:
  a) Soil Temperature: A linear regression model incorporating SSP-specific air temperature projections from KMA.
  b) Soil Respiration: An exponential regression model based on the relationship between soil temperature and respiration rates, calibrated using the measured data. The temperature sensitivity coefficient was derived using the Scipy library's curve-fitting function.

The projected data provide critical insights into potential changes in soil carbon fluxes under varying climate scenarios (Table 1).

**Table 1:** Overview of dataset structure: measured, estimated, and projected soil data The table summarizes the dataset structure, detailing measured data (2009–2010), estimated data (2011–2020), and projected data (2021–2100) under Shared Socioeconomic Pathway (SSP) scenarios. Each category includes descriptions of the data type, time period, and variables provided.

Folder	Files	Description
Measured Data (2009-2010)		
	Excel Sheet: “Measured Soil Temperature”	Monthly average soil temperature (°C)
	Excel Sheet: “Measured Soil Respiration”	Monthly average soil respiration (mg CO₂ m⁻² h⁻¹)
Estimated Data (2011-2020)		
	Excel Sheet: “Estimated Soil Temperature”	Monthly average soil temperature (°C)
	Excel Sheet: “Estimated Soil Respiration”	Monthly average soil respiration (mg CO₂ m⁻² h⁻¹)
Projected Data (2021-2100)		
	Excel Sheet: “Projected Soil Temperature”	Monthly average soil temperature (°C) for SSP1-2.6 to SSP5-8.5
	Excel Sheet: “Projected Soil Respiration”	Monthly average predicted soil respiration (mg CO₂ m⁻² h⁻¹) for SSP1-2.6-SSP5-8.5 scenarios

## 4 Experimental Design, Materials and Methods

**1. Study Area**

**Fig. 1:**
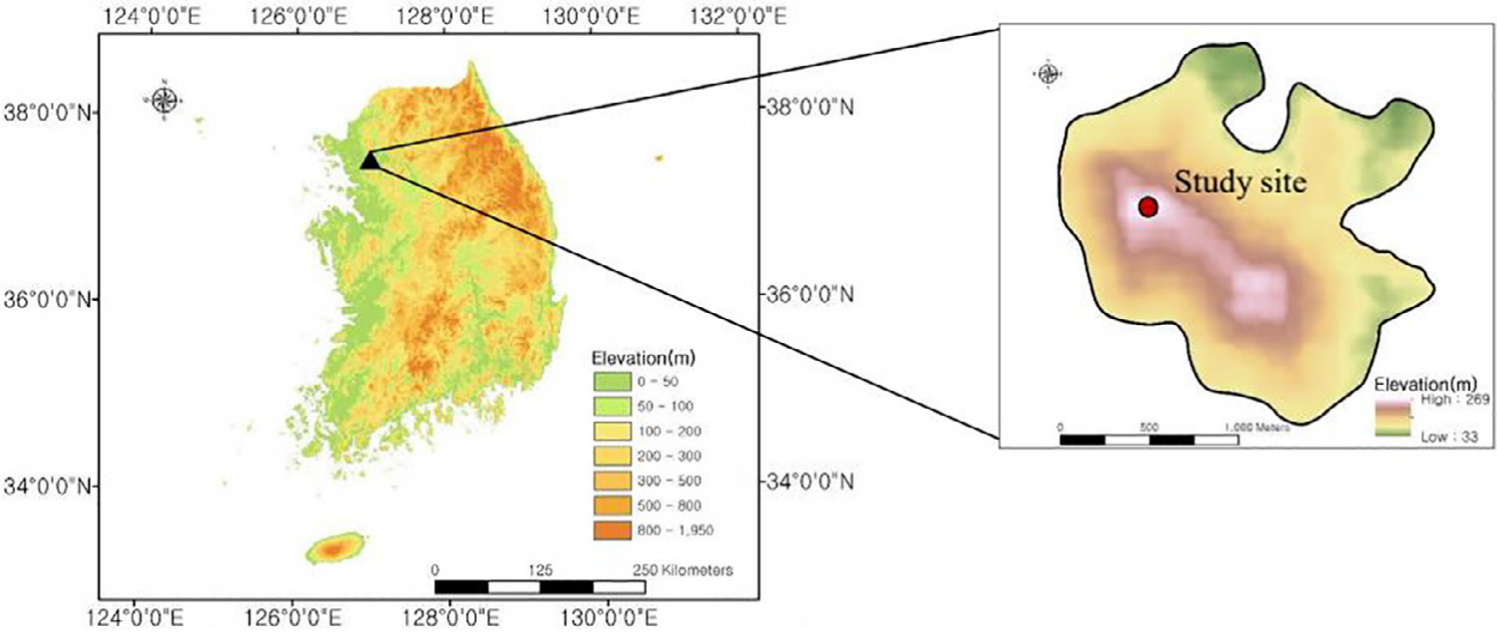
Location and topoghraphic map of Mt. Namsan study area in South Korea.

The study was conducted in the Quercus mongolica forest at Mt. Namsan, South Korea (37°32′53.5″ N, 126°59′40.1″ E), characterized by temperate forest ecosystems and distinct seasonal variations in temperature and precipitation. The study site is situated at an elevation of 205 m, with a northeast-facing slope (NE70°) and an inclination of 26° (Table 2). *Quercus mongolica* is the dominant species, with an average diameter at breast height (DBH) of 23.6 cm. The annual mean temperature is 11.8°C, and total annual precipitation averages 1,369 mm. This site represents typical temperate forest conditions, providing a valuable environment to examine soil respiration dynamics influenced by environmental changes [8] (Fig. 1).

**2. Soil Respiration Measurement (2009-2010)**

**Table 2:** Geographic and vegetative characteristics of the study area.

Latitude	37°32′53.5″ N
Longitude	126°59′40.1″ E
Elevation	205 meters
Aspect	NE70°
Slope	26°
Dominant species	*Quercus mongolica*
DBH	23.6 cm

Soil respiration and temperature were measured monthly from January 2009 to December 2010 using the closed chamber method [9,10]. Six cylindrical collars (16 cm in diameter, 12 cm in height) were installed 5 cm deep in the soil and secured to ensure airtight conditions. Measurements were taken using a Vaisala GMT-222/343 CO₂ sensor, with CO₂ concentrations recorded every 5 minutes over a 20-minute period. The soil respiration rate (RsRsRs) was calculated as:

$$Soil\;respiration\;\left(mg\;CO_{2}\;m^{-2}\;h^{-1}\right)\;=\;\alpha \cdot \rho \cdot \frac{V}{S}$$

Where:

*α* is the rate of increase in CO₂ concentration inside the collar (ppm min⁻¹),
*ρ* is the CO₂ density (mg m⁻³),
*V* is the volume of the chamber (m³),
*S* is the soil surface area covered by the collar (m²).

Atmospheric pressure and soil temperature (5 cm depth) were measured using a 7010 Pocket altimeter/barometer and Testo-925 thermometer, respectively. Soil moisture was monitored at 0–5 cm depth using a time-domain reflectometry (TDR) probe to assess its role in regulating soil respiration [1].

**3. Soil Temperature Estimation (2011-2020)**

Soil temperature data for 2011–2020 were estimated using a linear regression model based on the measured data from 2009–2010. The model incorporated air temperature data from the Korea Meteorological Administration (KMA) to predict soil temperature trends. The regression equation was expressed as:

$$T_{soil}=\beta_{0}+\beta_{1}\cdot T_{air}$$

Where:

*T_soil_* is the predicted soil temperature (°C),
*T_air_* is the air temperature (°C),
*β_0_* is the intercept,
*β_1_* is slope from the linear regression model.

This method was validated using an R² value of 0.85 and RMSE of 0.73°C, ensuring reliable estimations [2].

**4. Soil Temperature and Repiration Projections (2021-2100)**

a) **Climate Scenarios**
  The projections for 2021–2100 were based on SSP scenarios, as outlined in the Intergovernmental Panel on Climate Change (IPCC) Sixth Assessment Report (AR6) [11]. These scenarios describe distinct global trajectories for socioeconomic development, energy use, and greenhouse gas emissions. The four SSP scenarios represent a range of potential futures and were provided by the Korea Meteorological Administration (KMA). A brief description of each scenario is as follows:
  (1) SSP1-2.6 (Sustainability): A low-emission scenario focusing on sustainable growth through global cooperation, renewable energy adoption, and significant climate mitigation efforts. This scenario limits global warming to approximately 1.5–2°C by 2100, representing the best-case outcome.
  (2) SSP2-4.5 (Middle of the Road): A medium-emission scenario assuming moderate global progress, where historical socioeconomic and technological trends continue. Global warming is expected to stabilize at 2.5–3°C by the end of the century.
  (3) SSP3-7.0 (Regional Rivalry): A high-emission scenario characterized by regionalized economies, limited technological advancement, and continued reliance on fossil fuels. Global warming in this pathway reaches 3.5–4°C, with significant challenges to global climate action.
  (4) SSP5-8.5 (Fossil-Fueled Development): A very high-emission scenario driven by rapid economic growth and a strong reliance on fossil fuels, with minimal climate mitigation efforts. This scenario results in extreme global warming of 4–5°C by 2100, reflecting the worst-case outcome.
b) **Soil Temperature Projections**
  Air temperature projections for each SSP scenario were integrated into the linear regression model described above to estimate soil temperature changes for 2021–2100.
c) **Soil Respiration Projections**
  Projected soil temperatures were used to estimate soil respiration rates using an exponential regression model:
  $$R_{s}=R_{0}\cdot e^{\beta \cdot T_{soil}}$$

Where:

*R_s_* is the predicted soil respiration rate (mg CO₂ m⁻² h⁻¹),
*R_0_* is the baseline respiration rate,
*β* is the temperature sensitivity coefficient,
*T_soil_* is the predicted soil temperature (°C).

The *Q*₁₀ value, representing the proportional increase in respiration for every 10°C rise in soil temperature, was calculated to assess the temperature sensitivity of soil respiration [12].

**5. Data Analysis and Reproducibility**

Data analyses were conducted using IBM SPSS Statistics and standard statistical techniques. Descriptive statistics, including mean and standard deviation, were used to summarize soil respiration and environmental variables. Multiple regression models were employed to analyze the combined effects of soil temperature, moisture, and organic matter on respiration rates [13]. Seasonal trends were evaluated to identify significant patterns in soil respiration dynamics. Missing data during frozen soil conditions were interpolated using established temperature-respiration relationships [14]. All analytical methods and regression parameters are documented in the supplementary materials to ensure reproducibility.

## Limitations

This study has several limitations related to its geographic coverage, temporal range, and methodological constraints:

1. Geographic and Spatial Limitations
  The dataset is derived from a single site within the *Quercus mongolica* forest at Mt. Namsan, South Korea. Although this site is representative of temperate forest ecosystems, it does not account for variations across different altitudes, soil types, or forest structures. For example, comparative studies at higher-altitude sites, such as Mt. Jirisan, have shown significant differences in soil respiration dynamics due to cooler temperatures and increased organic matter accumulation [[1], [8]]. Future research should incorporate multiple sites to capture the spatial heterogeneity of soil carbon fluxes.
2. Temporal Range
  The measured data only cover a short two-year period (2009–2010), which may not adequately represent interannual variability in soil respiration and environmental conditions. Although estimated and projected data extend the temporal range, these rely on regression models that inherently simplify complex ecological interactions. Long-term measurements are needed to validate the predicted trends and to better understand how soil respiration responds to extreme climatic events or interannual climate variability [13].
3. Modeling Assumptions
  The linear and exponential regression models used for estimating and projecting soil temperature and respiration are based on several assumptions, including the linearity of air-to-soil temperature relationships and the temperature sensitivity of respiration. These models may not fully capture non-linear interactions between environmental factors, such as the interplay between soil moisture and temperature, which has been shown to have significant effects on respiration rates under certain conditions [3,14]. Incorporating more complex models, such as machine learning or process-based simulations, could improve the accuracy of future projections.
4. Uncertainty in Climate Scenarios
  The projections for 2021–2100 are based on Shared Socioeconomic Pathways (SSP) climate scenarios, which introduce a degree of uncertainty due to their reliance on assumptions about future socioeconomic and emissions pathways. For example, differences between SSP1-2.6 and SSP5-8.5 scenarios highlight the sensitivity of the projections to input variables. Further studies could explore a broader range of scenarios or integrate ensemble modeling to quantify projection uncertainties.
5. Environmental and Biotic Factors
  While this study focuses on soil temperature and respiration, other factors such as soil organic matter quality, microbial community composition, and plant-soil interactions were not explicitly measured or modeled. Previous research has demonstrated that these variables play critical roles in regulating soil carbon fluxes, particularly in ecosystems with high organic matter content or strong plant-microbial linkages [[8], [15]]. Including these variables in future studies could provide a more comprehensive understanding of the drivers of soil respiration.
6. Data Gaps and Missing Measurements
  During the 2009–2010 measurement period, certain environmental conditions, such as frozen soil during winter months, led to data gaps. Although interpolation methods were applied to address these gaps, the accuracy of these estimates may be limited, particularly for extreme conditions where standard models may not apply. Continuous year-round monitoring would help address this limitation and provide a more complete dataset.
7. Urban Influences
  The Mt. Namsan site is located within an urban area, which may introduce additional influences such as the urban heat island effect. This could impact soil temperature, moisture, and respiration rates, potentially limiting the generalizability of the findings to non-urban temperate forests. Comparative studies in non-urban *Quercus mongolica* forests would help isolate these effects and enhance the robustness of the conclusions.

## Conclusion

While the dataset provides valuable insights into soil temperature and respiration dynamics, these limitations underscore the need for multi-site, long-term, and interdisciplinary approaches to better understand soil carbon fluxes under changing climatic conditions. Future research should focus on addressing these gaps to enhance the accuracy and applicability of soil respiration models.

## Ethics Statement

This study did not involve any human or animal subjects. All data collection was conducted in compliance with ethical guidelines, and no personal data were used. The dataset consists of environmental measurements collected from the *Quercus mongolica* forest, and the research was conducted in accordance with the ethical standards of the National Institute of Ecology.

## CRediT authorship contribution statement

**Jae-Ho Lee:** Conceptualization, Methodology, Data curation, Writing – original draft. **Young-Ju Yu:** Conceptualization, Writing – original draft. **Yong-Su Kwon:** Writing – review & editing, Supervision. **Jae-Seok Lee:** Conceptualization, Methodology, Writing – review & editing, Supervision.

## Data Availability

EcoBankSoil Temperature and Respiration Datasetons under SSP Climate Scenarios (Original data)
EcoBankSoil Temperature and Respiration Datasetons under SSP Climate Scenarios (Original data)
